# Characterization of early myocardial inflammation in ischemia-reperfusion injury

**DOI:** 10.3389/fimmu.2022.1081719

**Published:** 2023-02-06

**Authors:** Qihong Wu, Rong Xu, Kun Zhang, Ran Sun, Mengxi Yang, Kuan Li, Hanrui Liu, Yiyuan Xue, Huayan Xu, Yingkun Guo

**Affiliations:** ^1^Department of Radiology, Development and Related Diseases of Women and Children Key Laboratory of Sichuan Province, West China Second University Hospital, Sichuan University, Chengdu, Sichuan, China; ^2^Department of Radiology, West China Second University Hospital, Sichuan University, Chengdu, Sichuan, China; ^3^Department of Radiology, Sichuan Cancer Hospital, Chengdu, Sichuan, China; ^4^Department of Prosthodontics, West China Hospital of Stomatology, Sichuan University, Chengdu, China

**Keywords:** myocardial ischemia reperfusion injury, MRI, inflammation, edema, apoptosis

## Abstract

**Background:**

Myocardial injury may be caused by myocardial ischemia-reperfusion (IR), and salvaging such an injury is still a great challenge in clinical practice. This study comprehensively characterized the physiopathologic changes of myocardial injury after IR to explore the underlying mechanism in the early reperfusion phase with particular emphasis on early myocardial inflammation.

**Methods and Results:**

The experimental IR model was obtained by the left anterior descending artery’s transient ligation of C57BL/6 mice. T2W signals of all mice showed increased signal at different IR stages. It was positively correlated with inflammatory cytokines and cells. T2W imaging by 7.0 T MRI surprisingly detected signal enhancement, but histopathology and flow cytometry did not reveal any inflammatory cells infiltration within 3 h after IR. Cardiomyocyte swelling and increased vascular permeability were observed by WGA staining and ultrastructural analysis, respectively. The 3 h IR group showed that the cardiomyocytes were severely affected with disintegrating myofilaments and mitochondria. Both VEGF and phosphorylated Src protein were markedly expressed in the 3 h IR group in comparison with the sham group, and TUNEL staining displayed little positive cells. Cleaved caspase-3 apoptin also has similar expression levels with that of the sham group. Resident macrophages had notably become M1 phenotype. The T2W signal was still elevated, and we observed that collagen deposition occurred from 1 to 7 days.

**Conclusions:**

The inflammation response during the first week after reperfusion injury gradually increase 3 h later, but the main manifestation before that was edema. This study indicated that the first 3 h may be crucial to the early rescue process for reperfusion-induced myocardial injury due to inflammatory cell infiltration absence and apoptosis.

## Graphical Abstract

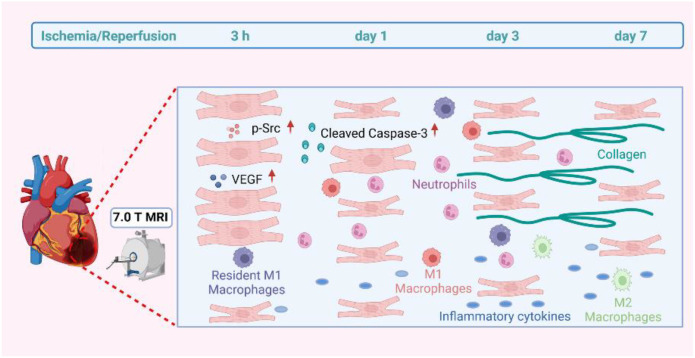

## 1 Introduction

Acute myocardial infarction (AMI) and the associated heart failure are the leading causes of death and disability worldwide (1, 2). Timely myocardial reperfusion using primary percutaneous coronary intervention (PCI) is the most effective treatment. However, the myocardial reperfusion process itself can induce cardiomyocyte death and myocardial injury, resulting in up to 50% final volume myocardial infarction (3). The ischemia-reperfusion (IR) injury rescue after reperfusion is still a great challenge in clinical practice, although the preferred reperfusion strategy time is within 2 h of ST-segment elevation MI diagnosis (4, 5).

The main mechanisms of myocardium IR injury involve inflammation, oxidative stress, mitochondrial damage, apoptosis, and autophagy (6, 7). Inflammation is significantly increased during reperfusion (8), which is activated at the stage of myocardial ischemia and plays a critical role in determining the AMI size and subsequent post-MI adverse left ventricular (LV) remodeling (9–12). The inflammatory process response includes inflammatory cell infiltration, and cytokine synthesis and secretion during myocardium IR (13). Previous findings have documented early neutrophils infiltration into the ischemia zone from 6 to 24 h post-AMI, followed by the accumulation of pro-inflammatory macrophages over the next 48-72 h, both of which contribute to cardiomyocyte death and myocardial IR injury (14). However, whether the inflammatory response was initiated in the very early reperfusion stage and its underlying mechanism still needs to be further investigated.

To recognize the myocardial pathophysiological changes *in vivo*, cardiac magnetic resonance imaging (MRI) has been identified as a promising imaging modality (15). And T2-weighted (T2W) imaging has particularly outstanding ability to determine edema with specific high signal (16) and has potential specificity for myocardial inflammation (17). Therefore, the present study focused on the myocardial inflammatory response and post reperfusion related tissue cellular changes in IR mouse model by 7.0 T MRI imaging and pathological analysis, spanning from the very early to late reperfusion stages, to provide new insight for myocardium rescue.

## 2 Methods

### 2.1 Mouse cardiac IR model induction

**Figure 1:**
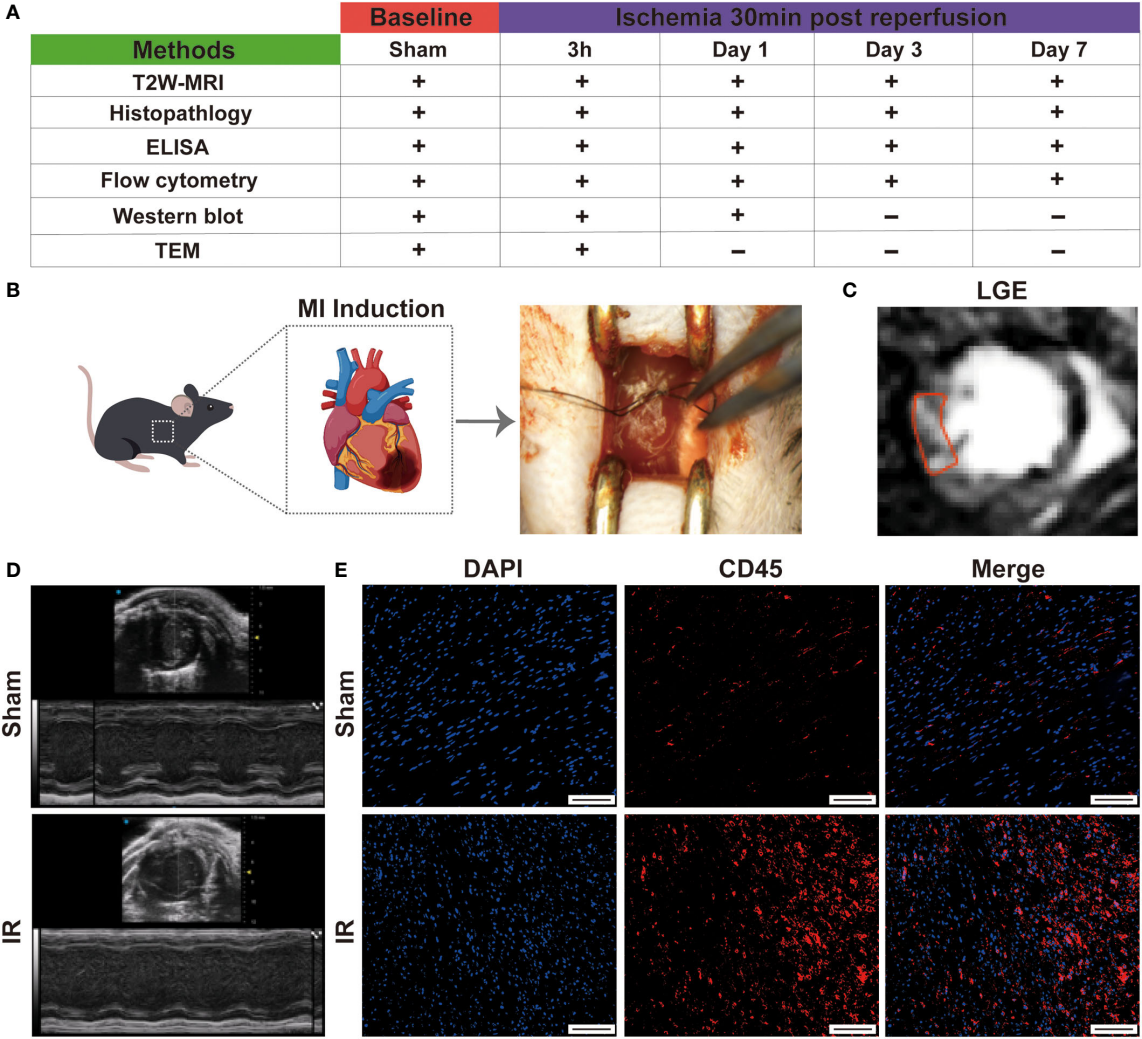
Establishment of mouse IR model. **(A)**, schema of *in vivo* protocol. The present study population comprised 60 mice which were used for the characterization of myocardial inflammation during the first week after IR. **(B)**, graphic image of myocardial IR injury induction. **(C)**, typical LGE image on day 7 in IR group. **(D)**, representative echocardiography on day 1 in sham and experimental group. **(E)**, immunofluorescence staining detect the infiltration of leukocytes (Bar=150 μm).

The experimental design is shown in **Figure 1A**. All animal procedure were approved by the Animal Experimentation Ethics Committee of West China Second Hospital of Sichuan University and conformed to the NIH Guide for the Care and Use of Laboratory Animals. C57BL/6 wild-type male mice of 8-10 weeks were used in this study. All mice were kept in the same room in a light-controlled environment with a 12:12 h light-dark cycle and with free access to standard mouse chow and water. The myocardial IR model was established as described in a previous study (18). Briefly, mice were anesthetized by isoflurane inhalation (1.5%-2%). Under a stereomicroscope, left thoracotomy was performed between the third and fourth ribs, and then the left anterior descending coronary artery was identified and ligated with a 8-0 polypropylene suture. A slipknot was tied over the vessel to create the occlusion. Ischemia was deemed successful when the myocardium supplied by the vessel turned pale. Mice of sham group were identically except that the ligature was not tied. After 30 minutes of ischemia, the slipknot was released by gently puling the slipknot sutures in opposite directions. At this time, reperfusion began and lasted for 7 days. All mice were euthanized by inhaling 3%-5% isoflurane.

### 2.2 MR imaging study

Magnetic resonance images were acquired using a NOVA 7.0 T preclinical horizontal MRI system (Time Medical Ltd). Mice were imaged on 3 h, day 1, day 3, day 7 after reperfusion. Baseline MRI scan was performed immediately before myocardial IR induction. All MRI images were acquired with ECG and respiration double-gating. Gated muilti-slice IntraGate Fast Low Angle Shot-cine was performed to confirm the heart position in three planes (short-axis, and 2- and 4-chamber long axis) to determine the location of LV. The echo signals of mice were measured with a T2W imaging sequence: TR = 800 ms, TE = 10.16 ms, slice thickness = 1.0 mm, matrix = 192 x 192, field of view = 40 x 40 mm, number of signal averages = 2. Successful IR induction was confirmed by late gadolinium enhancement (LGE) sequence (TRir = ~1 s depending upon respiratory rate, TE = 2.21 ms, slice thickness = 1.0 mm, matrix = 128 x 128, field of view = 40 x 40 mm, flip angle =907°) starting 15 min after intraperitoneal injection of 0.5 mmol/Kg Gd-DPTA. T2W signal and LGE area were evaluated using cvi42 software (Circle Cardiovascular Imaging) or ImageJ software.

### 2.3 Echocardiography

To assess LV physiology, echocardiography was performed using a Vevo 3100 system (VisualSonics, Toronto, ON, Canada) with a 30 MHz image transducer. Images were acquired prior to IR surgery (baseline) and at day 1 post-reperfusion. Mice were anesthetized with 1.5% isoflurane in an oxygen mix. Heart rate, body temperature, and electrocardiogram were monitored throughout the imaging procedure. Measurements were taken from the LV parasternal long axis (B-mode) and short axis (M-mode) views. For analysis, three images form consecutive cardiac cycles were included. Percent fractional shortening (FS) and ejection fraction (EF) were calculated as described previously (19).

### 2.4 Histological analysis

At indicated time points, hearts were fixed through trans-cardiac perfusion with saline followed by 4% paraformaldehyde (PFA), subsequently, the heart samples were immersed into 4% PFA and fixed over 24 h. Latter, the heart samples were embedded into paraffin blocks and cut into 5 μm thickness sections. Hematoxylin Eosin (H&E) staining, Masson trichrome staining (Solarbio ^®^) and WGA staining (VectorLabs) were performed following a standard protocol. The cell apoptosis was detected by using TUNEL System (Roche) according to the operating manual, after which the sections were incubated with DAPI for nucleus staining. For immunohistochemistry staining, the sections were rehydrated and heat-mediated antigen retrieval was performed using Target Retrieval Solution (S1699, Dako). The sections were incubated with 3% H_2_O2 (Sigma) to block endogenous peroxidase activity, followed by blocking with normal serum. A primary antibody specific for macrophages (CD68, Abcam; 1:400) was used at 4°C overnight, followed by incubation with rabbit anti-rat IgG and ABC reagent (VectorLabs). For immunofluorescence staining, the sections were blocked with goat serum at room temperature for 1 h. The primary antibodies (CD45, Servicebio; F4/80, Abcam; Ki67, Abcam) diluted in goat serum were then dropped to cover sections and incubated overnight at 4°C. Then the sections were washed three times with PBS, followed by incubating with a second primary antibody (Alexa Fluor 594, Abcam; Alexa Fluor 488, Life). DAPI was used for nucleus staining. Images were acquired on the Olympus IX73 imaging microscope. All histological analysis were quantified using ImageJ software.

### 2.5 Ultrastructural analysis by transmission electron microscopy

Cardiac tissue was prepared from sham group and 3 h group following reperfusion, and the infarct regions were sectioned. Tissue was fixed in 0.1 M sodium cacodylate buffer (pH 7.3) containing 4% paraformaldehyde and 1.5% glutaraldehyde for 2 hours, transferred to 5% glutaraldehyde overnight, then to 1% osmium tetroxide for 1 hour. Blocks were washed, dehydrated in a graded ethanol series, and embedded in Epon/Araldite resin. Ultrathin sections were stained with uranyl acetate and lead citrate and were viewed using a Hitachi HT7700 transmission electron microscope.

### 2.6 Cell preparation for flow cytometry

Samples were obtained from Left ventricular at indicated time points. Single cell suspensions were prepared as described previously, with some modifications (20). Briefly, hearts were cut into small pieces and digested with 0.3 mg/ml collagenase II (Invitrogen, USA), 0.3 mg/ml dispase II (Sigma, USA), DNase I (Biosharp, China) and 2.5 mM Cacl_2_ (Mackin, china) in HBSS solution (Invitrogen, USA) for 45 min at 37°C with gentle agitation. After the digestion, primary cardiac cells were obtained using Percoll (Solarbio ^®^) gradient separation and passed through 70-μm cell strainer. The obtained cells were washed with RPMI-1640 cell culture medium for further analysis.

### 2.7 Flow cytometric analysis

To block the nonspecific binding of antibodies to Fcγ receptors, isolated single cell suspensions were incubated first with anti-CD16/32 antibody (101302, Biolegend) at 4°C for 10 min. Subsequently, the cells were incubated with a mixture of antibodies at 4°C for 25 min. Anti-CD11b-PE (Cat: 101208, Biolegend), anti-Gr1-FITC (Cat: 108406, Biolegend), anti-F4/80-BV421 (Cat: 123132, Biolegend) and anti-CD206-Alexa674 (Cat: 565250, BD Pharmingen) were used for flow cytometric analysis. The obtained results were expressed as the percent. Flow cytometric data was analyzed using official FlowJo software.

### 2.8 Western blotting analysis

Left ventricles were homogenized in ice-cold RIPA lysis buffer containing 1% PMSF. The protein concentration was determined using Bradford BCA method (Beyotime, China). Total protein (30 μg) of each samples was separated by SDS-PAGE, transferred to 0.2 μm PVDF membrane (Millipore, USA) and probed with primary antibodies against VEGF (Cat: ET1604-28, HUABIO), p-Src (Cat: ET1609-15, HUABIO), Cleaved-caspase 3 (Cat: ab32042, Immunoway), CCL2 (Cat: HA500267, HUABIO) and GAPDH (Cat: ET1601-4, HUABIO). The ECL system was used for detection. GAPDH was used as an internal control and results expressed as the mean value.

### 2.9 ELISA assay

Blood samples were collected from the ophthalmic vein and kept 4 °C overnight before centrifugation for 15 min at 1000x g. Levels of IL-1β、TNF-α、TGF-β and Arg-1 in serum were measured with mouse ELISA kits (All from Animalunion, China) according to the manufacture’s instruction respectively.

### 2.10 Statistical analysis

The mice were randomized to experimental groups. All statistical data analyses were conducted using GraphPad prism 9.3.1 software. All experiments were performed independently at least three times, and the results were presented as the mean ± SD. Tow group means were compared by two-tailed independent samples student’s *t*-tests, while means of more than two groups were compared by one-way ANOVA. Correlation analysis was performed using Pearson’s or Spearman’s method, as appropriate. For all comparisons, *p < 0.05, **p < 0.01 and ***p < 0.001.

## 3 Results

### 3.1 Establishment of IR model in mice

The study design is showed in **Figures 1A, B**. Firstly, to evaluate the success of the model, we measured the cardiac function by performing echocardiography on day 1 post- myocardial IR induction. Following IR, the experimental mice displayed cardiac dysfunction and left chamber dilation, while the sham group exhibited a normal physiology (**Figure 1D**). Moreover, left ventricular EF and FS were significantly reduced in the experimental mice, as compared to those of the sham group (**Figure S1**). After 7 days, late gadolinium enhancement (LGE) magnetic resonance imaging revealed an obviously high light signal intensity in the experimental mice (**Figure 1C**), indicating the presence of cardiomyocyte necrosis. The infiltration of leukocytes is also a sign of successful modelling. Subsequently, the CD45 immunofluorescence staining revealed that more inflammatory cell infiltration was observed in the IR group (**Figure 1E** and **Figure S2**). These results suggested that the IR mice models were successful and could be used for subsequent experiments.

### 3.2 MRI evaluation of myocardial inflammation during the first week after IR

**Figure 2:**
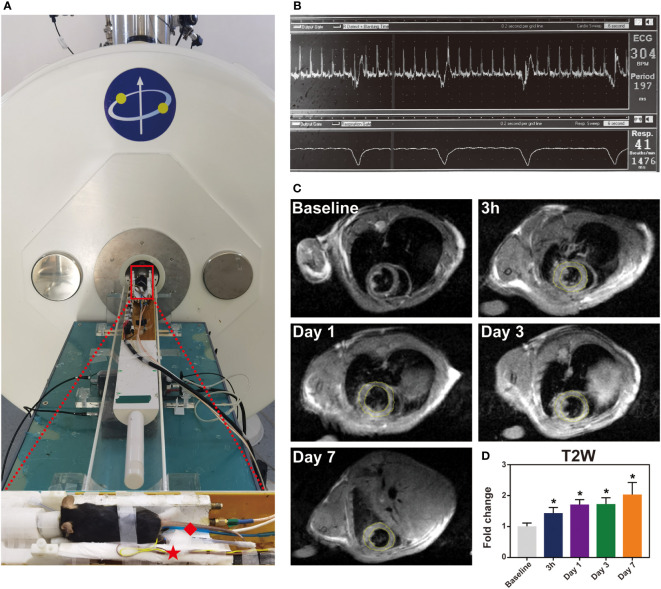
The immune response induced by IR injury at the first week. Representative images of histological changes in the ischemic myocardium. **(A)**, for each time point, images are shown of staining with H&E (Bar=1mm, 50μm). **(B)**, macrophages in mice post-IR were evaluated by CD68 staining (Bar=50 μm). **(C-F)**, plasma levels of IL-1β **(C)**, TNF-α **(D)**, Arg-1 **(E)** and TGF-β **(F)** were also determined by ELISA (n = 3-5). *p < 0.05 VS. Sham.

Myocardial IR is characterized by inflammation, which contributes to myocardial injury. To noninvasively evaluate inflammation, cardiac MRI was used. We utilized a small animal 7.0 T MRI to detect myocardial inflammation post-IR, specifically at baseline and at 3 h, 1 day, 3 days and 7 days post-IR. All MRI scans were acquired with an electrocardiogram and respiration double-gating method (**Figures 2A, B**). Baseline T2W image was relatively black in the LV anterior wall (**Figure 2C**). Initial reperfusion (3 h) was associated with a significant increase in T2W signal above that of the baseline, which subsequently increased progressively, reaching peak values on day 7 (**Figure 2D**). **Figure 2C** shows a representative example of one mouse serially scanned at all time-points.

### 3.3 Inflammatory response induced by myocardial IR injury

**Figure 3:**
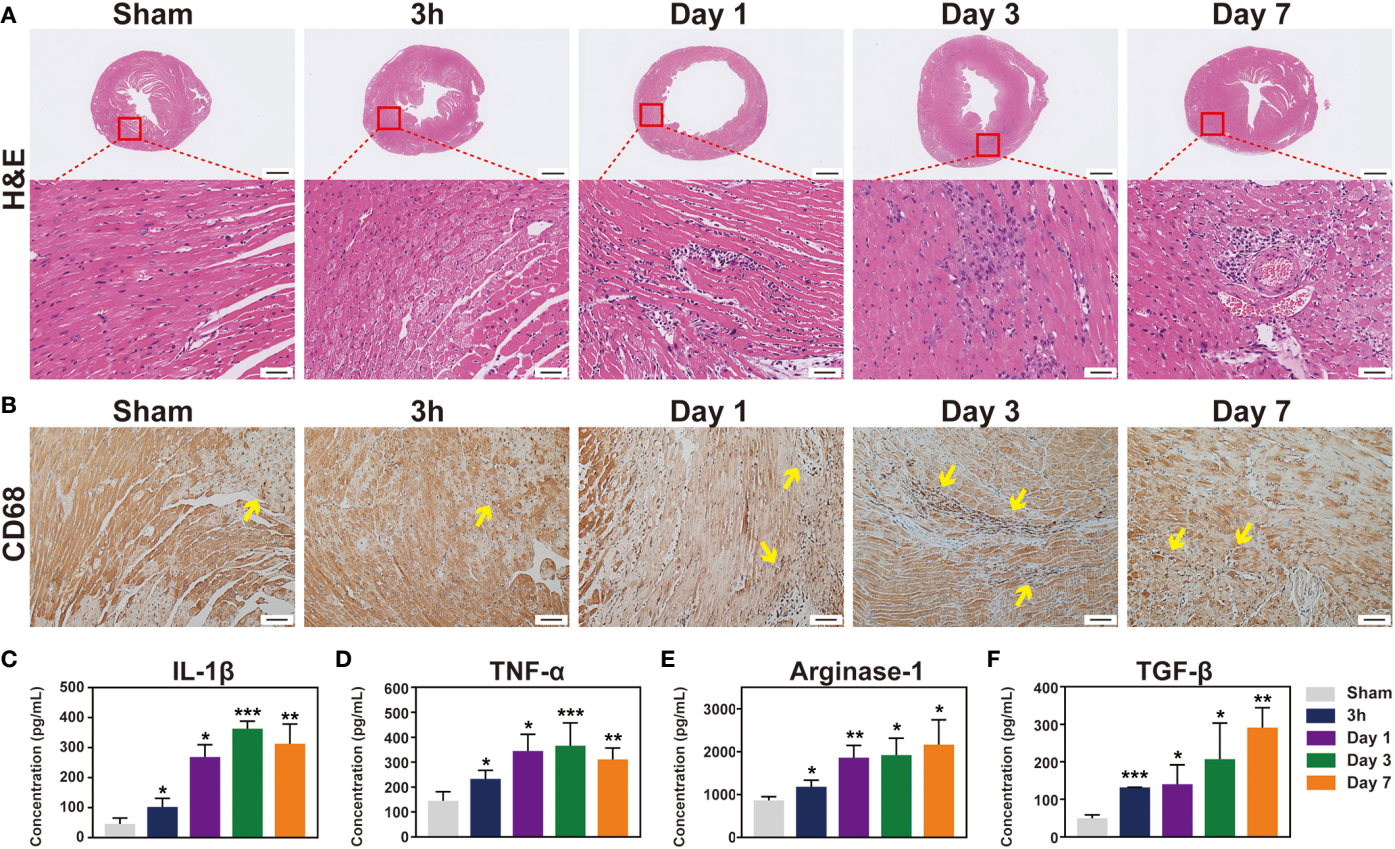
T2-weighted imaging sequence detect inflammation signal in the mouse heart during the first week post-IR. **(A)**, one mouse equipped with ECG (☆) and respiratory (◇) are being prepared to scan under 7.0 T MRI. **(B)**, real-time ECG and respiratory rate in a mouse. **(C)**, the typical T2W images from the same mouse before and after IR. **(D)**, quantitative the mean T2W signal intensity of the LV myocardium in each group, (n = 5). *p < 0.05 VS. Sham, ** p < 0.01, *** p < 0.001.

The inflammatory response after IR was subsequently evaluated *via* a histological analysis. H&E staining demonstrated an increase in leukocyte infiltration from day 1 to day 7 (**Figure 3A**). Monocyte/macrophage recruitment in the ischemic hearts was further investigated by immunostaining with CD68, and the number of infiltrated macrophages was evaluated (**Figure 3B** and **Figure S3**). Interestingly, the inflammatory cells before 3 h were absent, indicating that, although IR injury causes an inflammatory response, activated leukocytes have not reached the ischemic area yet in the very early stage (3 h). It is possible that increased macrophages may come from recruitment of peripheral monocytes or from proliferation of residential macrophages. As shown in **Figure S4A**, F4/80 and Ki67 were co-located in several cells (Person’s correlation coefficient: 0.14) at the site of myocardial ischemia after 1 day reperfusion, but this was absent in the sham group and post-reperfusion 3 h group. Additionally, we detected that CCL2 chemokine was highly expressed in post-reperfusion 1 day group (**Figure S4B**). At the same time, the ELISA results indicated that both anti-inflammatory cytokines IL-1 β and TNF-α and pro-inflammatory cytokines Arg-1 and TGF-β were improved after IR induction in the peripheral blood in mice (**Figures 3C-F**). Additionally, the expression level of myocardial IL-6 mRNA was significantly elevated in 3 h group (**Figure S5**). Collectively, these data indicated that the inflammatory response was induced and gradually increased after myocardial IR injury.

### 3.4 Temporal dynamic of the main cellular subsets of infiltration after IR

**Figure 4:**
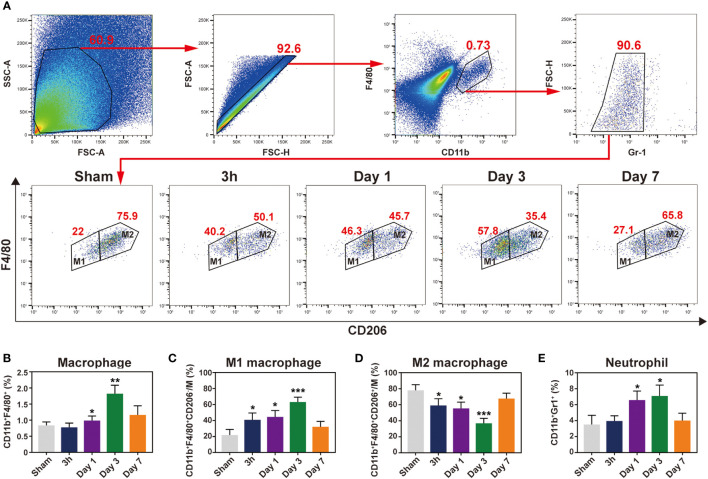
Characterization of temporal dynamic of the macrophages and neutrophil in the heart after IR. **(A)**, gating strategy for cardiac macrophages, M1 (CD11b^+^F4/80^+^Gr1^-^CD206^-^) and M2 (CD11b^+^F4/80^+^Gr1^-^CD206^+^) macrophages at indicated time point after myocardial IR injury. Represent flow cytometric images of M1 and M2 macrophages in the post-IR heart. **(B-E)**, the percentages of macrophages **(B)**, M1 **(C)**, M2 **(D)** and neutrophils **(E)** were determined in the hearts of sham group and reperfusion groups (n = 5-6, each). *p < 0.05 VS. Sham, ** p < 0.01, *** p < 0.001.

To detect major cellular components associated with inflammation, flow cytometry was conducted at 3 h and 1, 3, and 7 days following IR. Given the relatively low number of macrophages in the mouse heart, we used standard negative and positive magnetic beads (BD™ Comp Beads) to calibrate fluorescence compensation (**Figure S6**). The CD11b^+^ and F4/80^+^ double positive cells were defined as macrophages; pro-inflammatory M1 macrophages were labelled with CD206^-^ (CD11b^+^F4/80^+^Gr1^-^CD206^-^), whereas CD206^+^ was used to identify M2 macrophages (CD11b^+^F4/80^+^Gr1^-^CD206^+^) (**Figure 4A**). Neutrophils were defined as CD11b^+^Gr1^+^. After the IR injury, neutrophils accumulated in the ischemic heart, peaking at day 1 and then notably, continuing to accumulate in the myocardium over 3 days (**Figure 4E**). Moreover, macrophages began to infiltrate on day 1 and peaked on day 3 in the ischemic myocardium (**Figure 4B**), and these cells showed a biphasic pattern of activation. M1 macrophages increased gradually and dominated at 3 days post-IR, whereas the M2 macrophages represented the predominant cell subset after 3 days post-IR (**Figures 4C, D**). Consistent with the histopathological findings, flow cytometry also did not detect infiltrating inflammatory cells at 3 h after the IR injury. From these results, we found that neutrophils and macrophages were the major cellular components during the first week of IR injury, although inflammatory cell infiltration did not occur in the first 3 h post-IR.

### 3.5 Correlation of T2W signal with inflammatory components

**Figure 5:**
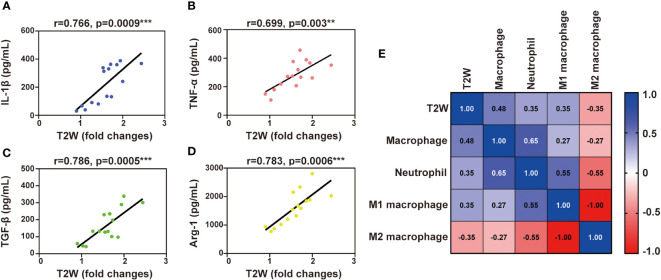
Liner correlation between myocardial T2W signal and inflammation mediator. **(A-D)**, scatter plot of myocardial T2W signal and IL-1β **(A)**, TNF-α **(B)**, TGF-β **(C)** and Arg-1 **(D)**. **(E)**, heat map of neutrophils, MI macrophages, M2 macrophages and macrophages among myocardial T2W signal, ** p < 0.01, *** p < 0.001.

To further prove whether T2W imaging can detect inflammation after myocardial IR in mice, a correlation analysis between T2W signal and inflammatory components was performed. Inflammatory cytokine activity in the serum of IR mice was significantly correlated with the T2W signal from 3 h to 7 days post-reperfusion (**Figures 5A-D**). In addition, both macrophage and neutrophil contents were also obviously correlated with the T2W signal. Interestingly, regarding macrophage phenotype, the pro-inflammatory M1 macrophages showed a moderate correlation with T2W signal rather than anti-inflammatory M2 macrophages (**Figure 5E**). These results indicate that T2W imaging could help detect myocardium inflammation in the IR mice models.

### 3.6 Myocardial edema occurs in the first 3 h after myocardial IR

**Figure 6:**
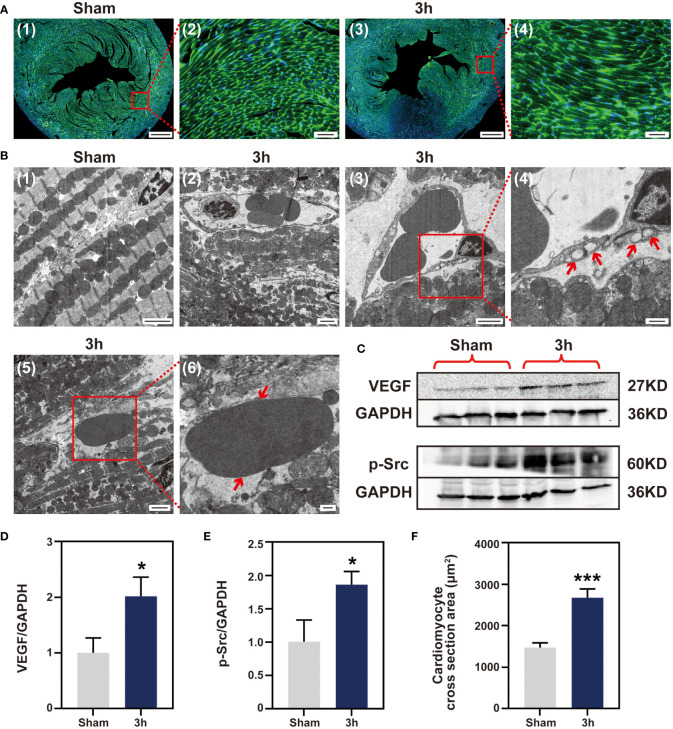
Myocardial IR induced edema at 3 h post-IR. **(A)**, WGA staining cardiomyocyte hypertrophy, (n = 3) (Bar=400 μm, 50μm). **(B)** (1-6), ultrastructural changes in mouse myocardium. (1), sham group ventricular myocardium showing normal myofilament architecture and mitochondria (Bar=2μm). (2), anomalous myofilaments and mitochondria are displayed after IR 3 h (Bar=2μm). (3-4), Vessel with no apparent gaps, but several large vacuoles apparent (Bar=2μm, 500nm). (5-6), section transverse to myocardium showing an RBC in the extracellular space (Bar=2μm, 500nm). **(C)**, western blotting detect VEGF and p-Src protein expression, (n = 3). Quantitative of VEGF protein **(D)**, p-Src protein **(E)** and cardiomyocytes area **(F)**. RBC, red blood cell; VEGF, vascular endothelial growth factor; p-Src, phosphor-Src, *p < 0.05 VS Sham, ***p < 0.001.

Considering the signal enhancement detected by T2W imaging, histopathology and flow cytometry did not reveal any infiltration of inflammatory cells during the first 3 h after reperfusion. Therefore, we turned our attention to myocardial edema. Firstly, the cross-sectional area of cardiomyocytes at the ischemia zone was determined by WGA staining, and the 3 h IR group showed a marked cardiomyocyte expansion compared with the sham group (**Figures 6A, F**). And the myocardial water content in 3 h group was higher than sham group (**Figure S7**). Subsequently, we evaluated the cardiac tissues at the ultrastructural level at 3 h after reperfusion. In the sham group, the section transverse to cardiomyocytes showed normal myofilament architecture and mitochondria (**Figure 6B-1**). In contrast to the normal myocardial tissue, the cardiomyocytes in the 3 h IR group were severely affected with disintegrating myofilaments and mitochondria (**Figure 6B-2**). At the same time, the blood vessels appear to be damaged and contained many large vacuoles (**Figure 6B-3**). Moreover, extravasated red blood cells were present in the interstitium (**Figure 6B-5**), which apparently escaped from the nearby vessels. The vascular endothelial growth factor (VEGF), first described as the “vascular permeability factor”, likely contributes to the development of myocardial edema (21), and VEGF-mediated Src signaling had been proved to be involved in disease progression following MI (22). To further explore the possible mechanism of IR-induced edema, we evaluated the expression of VEGF-mediated Src signaling pathway. As shown in **Figures 6C-E**, in comparison with the sham group, both VEGF and phosphorylated Src proteins were markedly expressed in the 3 h IR group. These data showed that edema obviously appear at 3 h post-IR, and was probably mediated by the VEGF-Src signaling pathway.

### 3.7 No cell apoptosis occurs at 3 h post-IR

**Figure 7:**
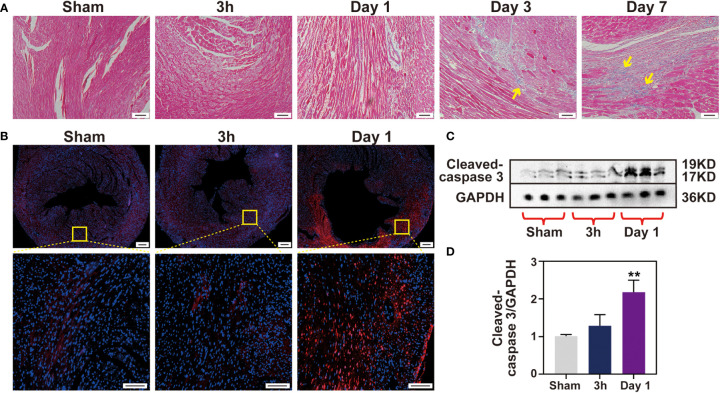
No cell apoptosis happens after 3 h IR. **(A)**, Masson staining of heart sections was performed for each time point (Bar 100 μm). **(B)**, cell apoptosis was measured by TUNEL assay (Bar =200 μm, 50μm) and Western blotting **(C)** in sham group and 3 h, 1 day after IR induction. Quantitative of cleaved-caspase 3 protein expression level **(D)**, (n = 3). **p < 0.01 VS. Sham.

Myocardial cell apoptosis has been reported to contribute significantly to IR-induced myocardial injury, which motivated us to determine whether apoptosis occurs at the very early stage of injury. Firstly, detection of apoptosis by TUNEL staining was performed in ischemic tissue sections. Little TUNEL positive cells were found in the 3 h IR group. On the contrary, the number of TUNEL positive cells in the day 1 IR group was significantly increased when compared with that of the sham group (**Figure 7B** and **Figure S8**). Subsequently, the extracts of cleaved-caspase 3 apoptin isolated from the ischemic zone in the three groups were examined *via* Western blotting analysis. As shown in **Figures 7C, D**, the cleaved-caspase 3 protein in the 3 h IR group has similar expression levels with that of the sham group, although an obviously higher expression level was observed in the day 1 IR group. Likewise, caspase 3 mRNA expression in the 3 h group displayed no obvious difference with sham group (**Figure S9**). In addition, we found that collagen was gradually deposited in the ischemia myocardium post-IR (**Figure 7A** and **Figure S10**). The trend is similar to the performance of inflammatory cytokines, implying that IR-induced inflammatory response may be involved in the tissue healing processes.

## 4 Discussion

Characterization of the time course of the myocardium IR-induced inflammation and its determinants is critically important for the development of diagnostic and therapeutic applications. In the present longitudinal experiment study, we explored the inflammatory response of myocardial post-IR and the possible underlying mechanisms by performing a comprehensive histopathological cellular and advanced MRI serial analysis using a widely used small animal model.

MRI allows *in vivo* myocardial characterization and cardiac MRI has been identified as a promising imaging modality (15). T2W imaging techniques have been widely used to detect edema and are potentially specific for myocardial inflammation (17). Recently, our team has demonstrated that cardiac MRI could help detect inflammation in the remote myocardium in MI porcine models (23). In this study, we first utilized a 7.0 T magnetic resonance T2W imaging sequence to detect post- myocardial IR inflammation in a widely used small animal model. As expected, the T2W signals were positively correlated with inflammatory cytokines and cells. We found that the T2W signals were abnormal at 3 h of perfusion and then increased gradually, suggesting that myocardial edema or inflammation had started very early.

Macrophages and neutrophils secrete pro-inflammatory cytokines in the early stage of MI, which leads to sustained inflammation and myocardial injury (24). The neutrophils peak shifted from day 3 to day 1, while the macrophages infiltration peak switched from day 7 to day 3 in mice IR model, compared with permanent ligation MI (25). Reperfusion temporally shifted the innate immune cell to an earlier time point, indicating that timely reperfusion may benefit from ischemic myocardial recovery by preventing the transition from acute to persistent inflammation. Macrophages are composed of two populations, including pro-inflammatory M1 and reparative M2 macrophages. The M1 macrophages predominate at the early stage after myocardial IR, followed by a gradual increase of the M2 subset (26). Macrophages undergo a rapid shift to an M1 subset and elicit inflammatory cytokine secretion, lowering the CD206 expression. At 7 days after reperfusion, the M2 subset became the predominant macrophage. The pro-inflammatory M1 macrophages showed moderate correlation with the T2W signals rather than the anti-inflammatory M2 macrophages combined with our MR imaging results, suggesting that T2W imaging may tend to reflect pro-inflammatory cell infiltration.

Interestingly, there was no infiltration of inflammatory cells, including neutrophils and macrophages, at 3 h post-reperfusion. A previous study reported that the neutrophils in the myocardium subjected to 45 minutes of ischemia followed by 4 h of reperfusion significantly increased in comparison with the normal myocardium from the same hearts (27). Thus, we speculate that 3 h may be the critical time point for inflammatory cells to reach ischemic myocardium. It is reported that IL-6 expression is obvious after reperfusion 3 h (28). Indeed, we also detected IL6 changed obviously after 3 h reperfusion in the heart (**Figure S5**). This is consistent with our ELISA and T2W results that detected inflammation at 3 h reperfusion. After carefully examining our flow cytometry data, we found that the resident macrophages had already become M1 phenotype at 3 h post-reperfusion (**Figure S11**), despite the absence of infiltration of inflammatory cells, which not only contribute to the source of inflammatory cytokines (such as IL-1β and IL-6) but partly explain the enhancement of T2W signal. The changes in the phenotype of resident macrophages preceded the infiltration of inflammatory cells, suggesting that they may play a vital role in the initiation of myocardial reperfusion-induced injury. Owing to the abundance and phenotypic plasticity of macrophages, they are well fit for mediating the repair response after IR. Most of the previous studies have made great progress in the treatment of IR-induced injury by regulating the recruited macrophage polarization (29). According to our data, we believed that more attention should be paid to resident macrophages.

As with most organs, water is the primary component of cardiac tissues. In homeostasis, myocardial water is stable and nearly intracellular, with only a little interstitial component. In the context of MI, edema appears initially in the form of cardiomyocyte swelling during the early stages of ischemia (30). Myocardial edema is then obviously exacerbated upon reperfusion of blood flow to the ischemic region. This increase is due to the enhancement of interstitial edema, due to water permeability and protein leakage (31). Given that there was no infiltration of inflammatory cells, but rather a clear signal was detected on T2W imaging at 3 h post-reperfusion. We therefore shift our focus on myocardial edema. As expected, myocardial water content in the 3 h group was higher than sham group (**Figure S7**). Our results indicated that early edema was partly due to cardiomyocyte swelling and increased vascular permeability, while the intracellular and extracellular edema were undetermined. A method based on MRI has been developed to differentiate intracellular and extracellular myocardial water compartments, but the intracellular water distribution does not accurately reflect intracellular edema (32). Further studies are needed to discriminate between intracellular and extracellular edema. Notably, a comprehensive work has observed that the myocardial edema after IR is not stable and follows a bimodal pattern in the pig model (33). However, we did non see a drop in T2W signal during the reperfusion in a small animal model. The absence of bimodal in the mouse heart and the apparent presence of bimodal in the pig heart after IR may be associated with species differences.

After MI, an increase in VEGF levels occurs and contributes to detrimental myocardial edema (34). Genetics interferes with the ability of VEGF to mediate the increased vascular permeability, which is correlated with reduced LV edema in mice and improved survival after MI (35). Src is implicated as the tyrosine kinase responsible for phosphorylation of vascular endothelial-cadherin and the elevated vascular permeability (36). Constitutive Src gene inaction is accompanied by reduced edema and improved long-term outcome after MI (22). We thus concluded that the VEGF/Src-mediated pathway may be a key signal molecule in the very early reperfusion stages; however, additional works are needed, in particular, to assess whether a combined treatment of myocardium edema and inflammation can accelerate the recovery of heart function.

It has been investigated that myocardial edema induced by reperfusion may contribute to cell death. Cell apoptosis is obviously initiated at 6 h reperfusion, which progressively progressed into myocardial apoptotic cell death during the late phase of reperfusion in a canine model (37). Gottlieb et al. (27), however found that apoptosis was detected in the ischemia myocardium after 30 min of ischemia and 4 h of reperfusion in a rabbit model. In the current mouse model, we observed that there was no obvious cell apoptosis in the ischemic myocardium at 3 h of reperfusion. Given that apoptosis represents a potentially preventable form of cell death, identifying its timing may help in developing potential treatment strategies for alleviating myocardial IR injury by regulating apoptosis. Both inflammatory cell infiltration and apoptosis are involved in the early myocardial IR injury, and they greatly affect cardiac repair and healing. The data presented here implicate that the first 3 h of reperfusion, when inflammatory cell infiltration and apoptosis have not been initiated yet, may be crucial to perform rescue procedures for the ischemia myocardium.

In clinical practice, timely PCI is an essential step to rescue the ischemic myocardium to restore reperfusion in patients with ischemic cardiomyopathy. However, a substantial number of patients could experience severe myocardial IR injury and these complications may be associated with an increased risk of major adverse cardiac events (MACE). Therefore, early identification and myocardial IR injury intervention after PCI is an important reason for improving the prognosis. Recent clinical guidelines suggest that cTn monitoring should be performed at least once 3-6 h after PCI to determine the myocardial injury extent and determine subsequent management strategies (38). However, it remains a challenge for new treatment or appropriate time window to reduce the risk of MACE. This study comprehensively characterized the myocardial injury process after IR in mice *in vitro* and *in vivo*. It is feasible to develop new strategies to reduce the myocardial IR injury by targeting myocardial edema, resident macrophages, apoptosis and inflammatory cell infiltration collectively in the first 3 h after reperfusion. However, further interventions and population cohort studies are needed to clarify the importance of myocardial rescue within 3 h of IR injury due to the differences in animal experiments and clinical scenarios.

We proved that the inflammatory response during the first week after reperfusion gradually increase at 3 hours later, and before that, the main manifestation was edema probably induced by the activation of VEGF/Src signaling pathway. Meanwhile, the infiltrating inflammatory cells and apoptosis are absent in the very early reperfusion stage. These data reveals that the first 3 h of reperfusion may be the vital for ischemia myocardial recovery, especially when developing anti-inflammatory strategies. We investigated the critical time point for changes in cardiac pathophysiology in IR myocardium, a finding with potentially important implications for managing patients with reperfusion after MI.

## Data availability statement

The raw data supporting the conclusions of this article will be made available by the authors, without undue reservation.

## Ethics statement

The animal study was reviewed and approved by Animal Experimentation Ethics Committee of West China Second Hospital of Sichuan University.

## Author contributions

QW and RX: conceptualization, methodology, software. KZ, RS and MY: data curation, statistical. KL, HL and YX: visualization, experimental studies. HX and YG: manuscript preparation, reviewing and editing, validation. All authors contributed to the article and approved the submitted version.
