# Supplementary material for: Characterization of early myocardial inflammation in ischemia-reperfusion injury
Source: Front Immunol. 2023 Feb 6;13:1081719. doi: 10.3389/fimmu.2022.1081719 (PMC9939645; doi:10.3389/fimmu.2022.1081719)
Supplement: Supplementary file 1 [file DataSheet_1.docx]

**SUPPLEMENTARY FIGURES**


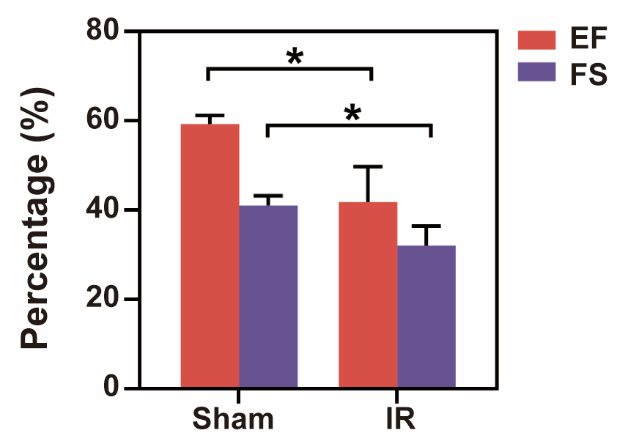


**Figure S1**. EF and FS were measured, n = 4. *p < 0.05 VS. Sham. EF, ejection fraction; FS, fraction shorting.


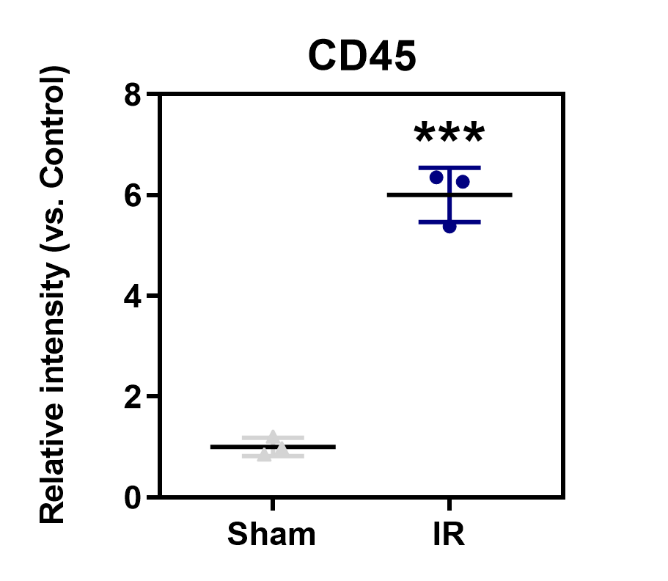


**Figure S2.** The quantitative analysis of CD45 immunofluorescent images was performed according to IOD (integrated optical density) by Image Pro Plus 6.0 software. Statistical analysis of the data was performed by using SPSS 23.0 software (SPSS Inc., USA). ***P<0.001, n=3.


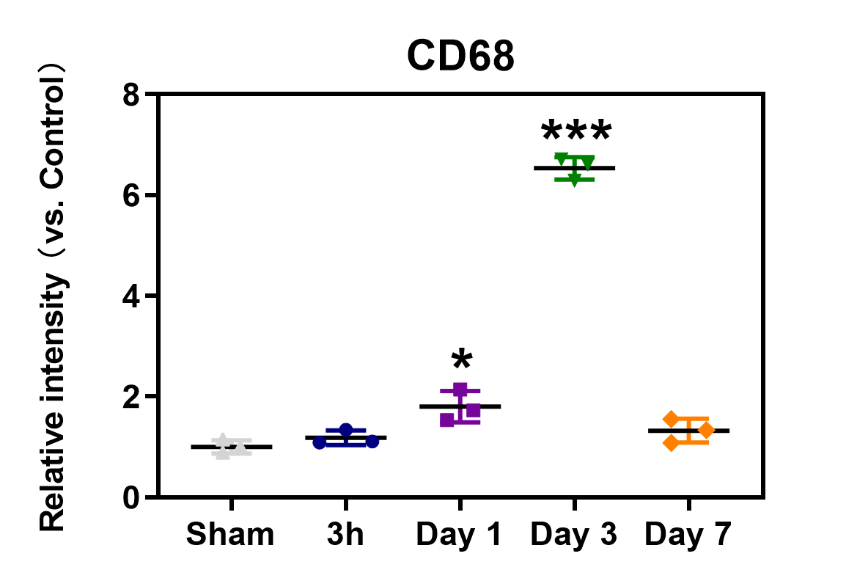


**Figure S3.** The quantitative analysis of CD68 staining images was performed according to IOD (integrated optical density) by Image Pro Plus 6.0 software. Statistical analysis of the data was performed by using SPSS 23.0 software (SPSS Inc., USA). ***P<0.001, n=3.


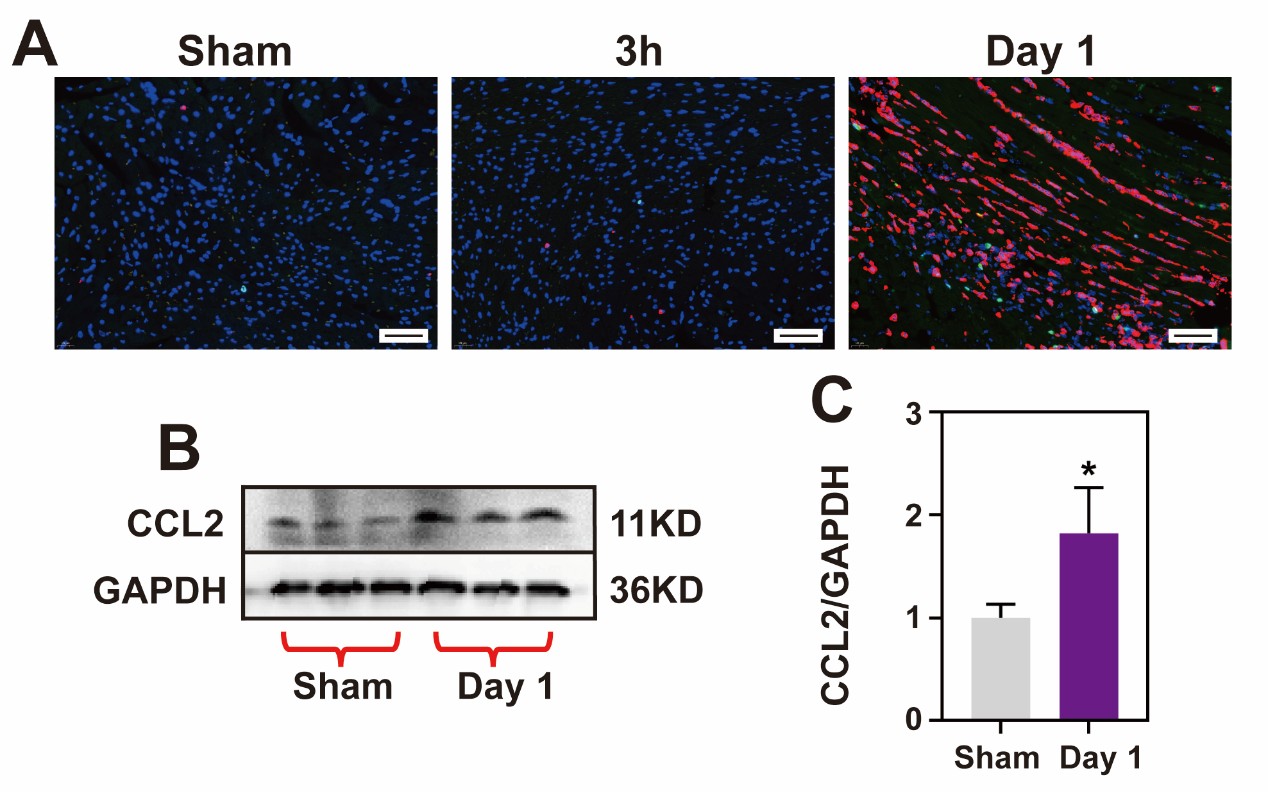


**Figure S4.** Monocytes recruitment was happened after reperfusion. A, the expression of F4/80 (Red) and Ki67 (Green) in the heart were detected by immunofluorescence. Bar = 40 μm. B, Western Blot detect CCL2 protein expression in the heart. *p<0.05, n=3.


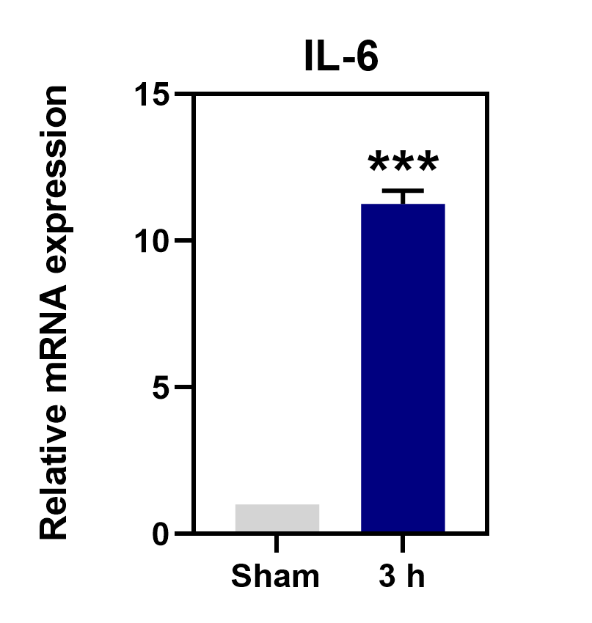


**Figure S5. qPCR evaluate mRNA expression of IL-6 in the heart, n=3.**


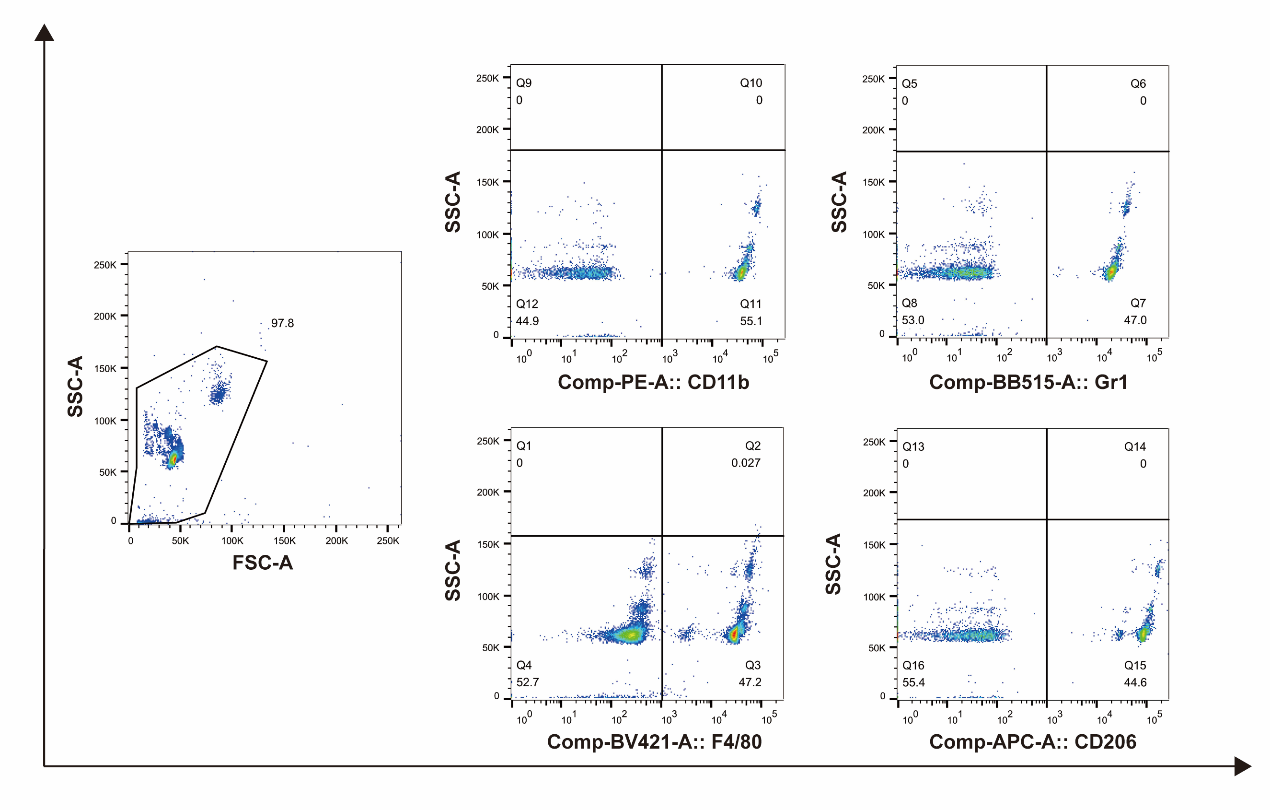


**Figure S6.** Represent flow cytometric images of magnetic beads.


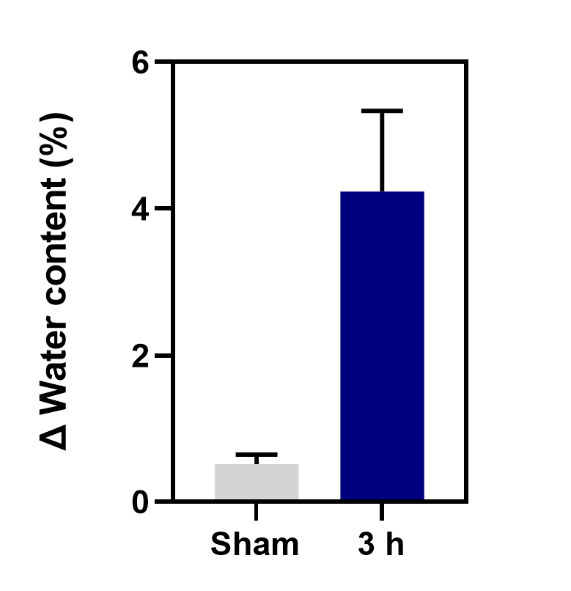


**Figure S7. Absolute differences (%) in myocardial water content between ischemia and remote zones, n=3.**


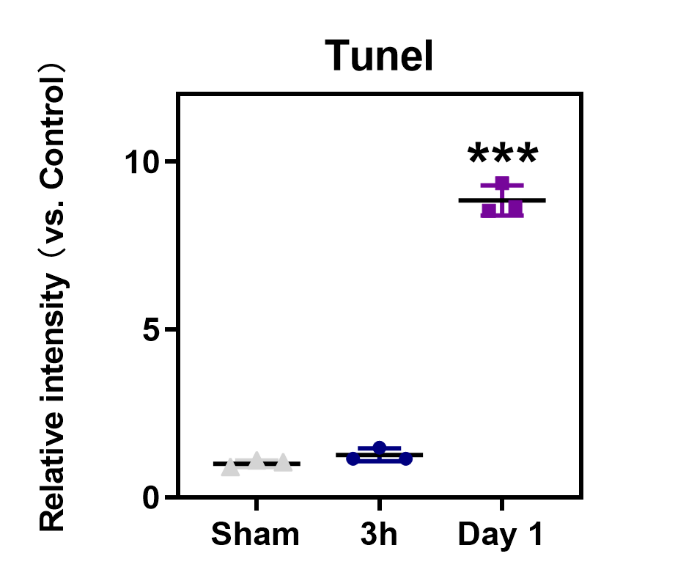


**Figure S8.** The quantitative analysis of TUNEL staining images was performed according to IOD (integrated optical density) by Image Pro Plus 6.0 software. Statistical analysis of the data was performed by using SPSS 23.0 software (SPSS Inc., USA). ***P<0.001, n=3.


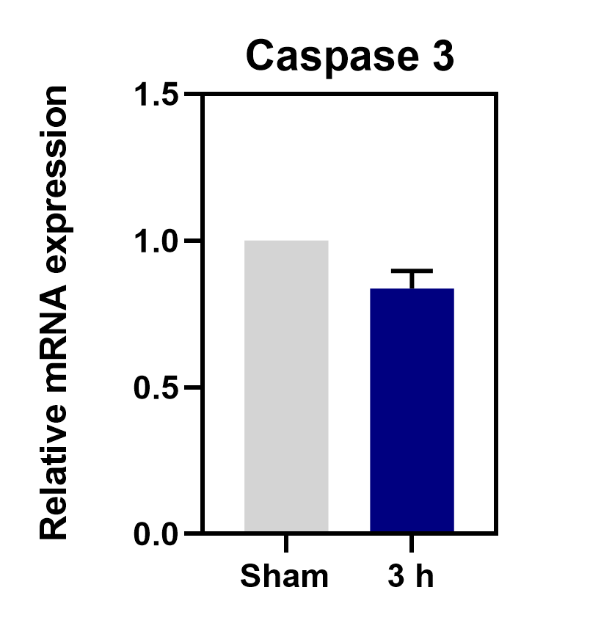


**Figure S9. Myocardial apoptosis was evaluated by qPCR analysis of mRNA expression of caspase 3 in the heart, n=3.**


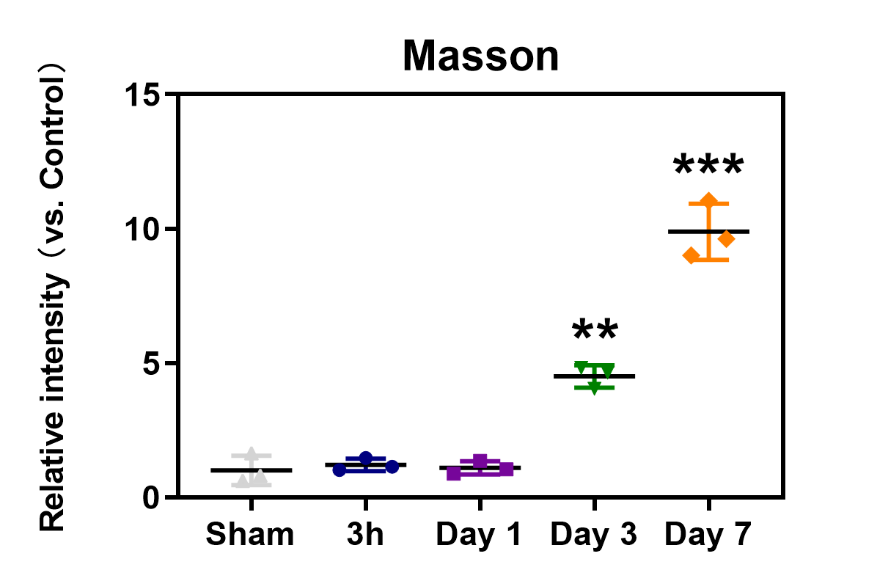


**Figure S10.** The quantitative analysis of Masson staining images was performed according to IOD (integrated optical density) by Image Pro Plus 6.0 software. Statistical analysis of the data was performed by using SPSS 23.0 software (SPSS Inc., USA), **p<0.01, ***p<0.001, n=3.


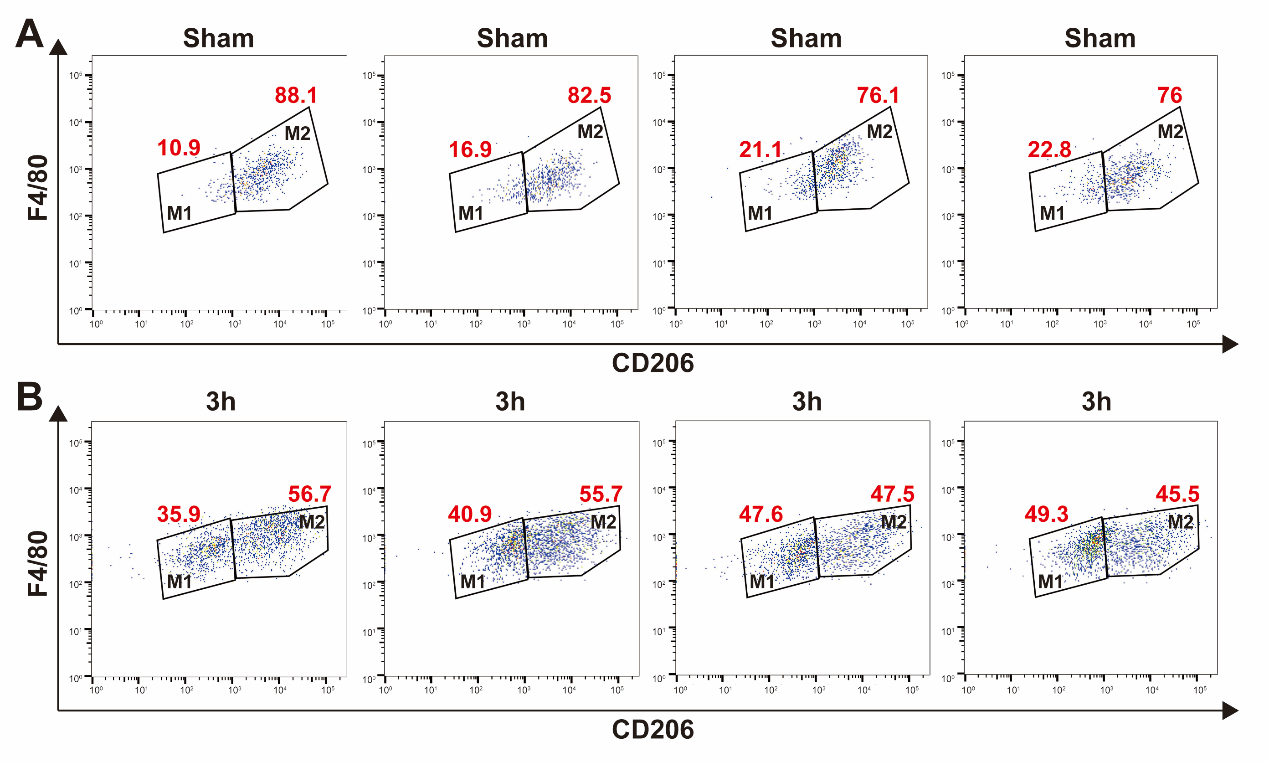


**Figure S11**. Represent flow cytometric images of M1 and M2 macrophages in the post-IR heart. A-B, the percentages of macrophages in sham group and 3 h group, respectively, n = 6.

**SUPPLEMENTAL TABLE**

**Supplemental Table 1. List of primers used for quantitative real-time PCR**

| Primer name | Sequences (5’-3’) |  |
| --- | --- | --- |
| m*GAPDH*-F  m*GAPDH*-R  m*IL6*-F  m*IL6*-R  m*Caspase3*-F  m*Caspase3*-R | GCAAGGACACTGAGCAAGAG  GGGTCTGGGATGGAAATTGT  ATGAAGTTCCTCTCTGCAAGAGACT  CACTAGGTTTGTTTAATCTC  AGTAAAGACCATACATGGGAGCA  AGCGAGATGACATTCCAGTGC |  |

**SUPPLEMENTAL METHODS**

**Quantitative real-time PCR**

Total RNA of mouse heart was purified by using TRIzol Reagent (Takara, Japan) and reversed -transcribed to cDNA with Prime Script TM RT Master Mix (Takara, Code No. RR036A) according to the manufacturer’s instructions. qPCR was performed with Applied Biosystems 7500 (Life Technologies corporations, USA). Relative gene expression IL-6 and caspase 3 were normalized to GAPDH using ΔΔCt method. The primer sequences are shown in Supporting Information Table S1.

**Quantification of myocardial water content**

Paired myocardial samples were collected within minutes of euthanasia from infarcted and remote myocardial of all mice. Tissue samples were immediately blotted to remove surface moisture and weighed on a high-precision scale. The culture dish were weighed before and after drying for 48 h at 80℃ in a desiccating oven. Tissue water content was calculated as follows: water content (%) = [(wet weight-dry weight)/wet weight] ×100.
